# Gentamicin Combined With Hypoionic Shock Rapidly Eradicates Aquaculture Bacteria *in vitro* and *in vivo*

**DOI:** 10.3389/fmicb.2021.641846

**Published:** 2021-04-06

**Authors:** Yuanyuan Gao, Zhongyu Chen, Wei Yao, Daliang Li, Xinmiao Fu

**Affiliations:** ^1^Provincial University Key Laboratory of Cellular Stress Response and Metabolic Regulation, Key Laboratory of Optoelectronic Science and Technology for Medicine of Ministry of Education, College of Life Sciences, Fujian Normal University, Fuzhou, China; ^2^Engineering Research Center of Industrial Microbiology of Ministry of Education, Fujian Normal University, Fuzhou, China; ^3^Fujian Key Laboratory of Innate Immune Biology, Biomedical Research Center of South China, Fuzhou, China; ^4^College of Life Science, Fujian Normal University, Fuzhou, China

**Keywords:** aquaculture bacteria, persister, antibiotic tolerance, aminoglycoside, gentamicin, neomycin, hypoionic shock, zebrafish

## Abstract

Bacterial pathogens are a major cause of infectious diseases in aquatic animals. The abuse of antibiotics in the aquatic industry has led to the proliferation of antibiotic resistance. It is therefore essential to develop more effective and safer strategies to increase the efficacy and extend the life span of the antibiotics used in aquaculture. In this study, we show that six aquaculture bacterial pathogens (i.e., *Aeromonas hydrophila*, *Vibrio alginolyticus*, *Edwardsiella tarda*, *Streptococcus iniae*, *Vibrio harveyi*, and *Vibrio fluvialis*) in the stationary phase can be rapidly killed after immersion in gentamicin- or neomycin-containing, ion-free solutions for a few minutes. Such hypoionic shock treatment enhances the bacterial uptake of gentamicin in an ATP-dependent manner. Importantly, we demonstrate, as a proof of concept, that gentamicin under hypoionic shock conditions can effectively kill *A. hydrophila in vivo* in a skin infection model of zebrafish (*Danio rerio*), completely curing the infected fish. Given that pathogenic bacteria generally adhere to the skin surface and gills of aquatic animals, our strategy is of potential significance for bacterial infection control, especially for small-scale economic fish farming and ornamental fish farming. Further, the combined treatment can be completed within 5 min with a relatively small volume of solution, thus minimizing the amount of residual antibiotics in both animals and the environment.

## Introduction

Aquaculture is the fastest-growing animal food industry at present and provides human society with one of the most sustainable forms of edible protein and nutrient production, making it a fundamental part of future food production (Froehlich et al., 2018). Similar to other animal production sectors, fish production relies on intensive and semi-intensive cultivations, which result in increased disease outbreaks (Little et al., 2016; Kotob et al., 2017). Fish diseases are often caused by bacteria, viruses, fungi, parasites, or a combination of these pathogens, with bacterial pathogens being the most common etiology (Dhar et al., 2014; Lafferty et al., 2015). Given that bacteria can survive well in aquatic environments independent of a host, bacterial diseases have become major impediments to aquaculture (Haenen et al., 2013). Various effective vaccines have been developed against many fish bacterial pathogens. Nevertheless, some infectious bacterial diseases that cannot be controlled using conventional inactivated vaccines are threatening aquaculture (Tafalla et al., 2013). In addition, attenuated bacterial vaccines can potentially revert to a pathogenic form, which poses a tremendous risk to the whole environment (Matsuura et al., 2019). Furthermore, some bacterial pathogens are difficult to culture or completely unculturable, making them unsuitable for vaccine development (Takano et al., 2016).

Antibiotics are widely used to prevent and control bacterial diseases in aquaculture (Cabello et al., 2016; Santos and Ramos, 2018). Nevertheless, long-term antibiotic usage, particular overuse and misuse, has led to the emergence and proliferation of antibiotic resistance (Baym et al., 2016; Du et al., 2019). Improving the efficacy of antibiotics is a promising strategy for extending the life span of current antibiotic drugs (Levy and Marshall, 2004; Nambiar et al., 2014). Previously, we found that treatment in non-electrolyte (e.g., glycerol) solutions or in ultrapure water exhibits a potentiation effect on the killing of bacteria by aminoglycosides, while treatment in strong electrolyte (e.g., NaCl) solutions barely exhibits any potentiation effects (Jiafeng et al., 2015). In particular, stationary-phase *Escherichia coli* cells can be killed after treatment in aminoglycoside-containing ultrapure water for only 1–2 min (Jiafeng et al., 2015). It should be noted that bacteria enter a non-growth state defined as a stationary phase when nutrients are insufficient in their living surroundings (Navarro Llorens et al., 2010), and stationary-phase bacteria are much more resistant than exponential-phase cells to destruction by antibiotics (Levin and Rozen, 2006; McCall et al., 2019). Furthermore, we showed that such hypoionic shock (i.e., the absence of ions) could dramatically potentiate the killing of nutrient shift- or starvation-induced *E. coli* persister cells by aminoglycosides in 3 min (Chen et al., 2019).

Here, we investigated whether this unique approach can kill aquaculture bacterial pathogens, given that bacterial pathogens usually infect fish by attaching to their skin surface, gills, and gut lining, which are always in intimate contact with the surrounding water (Benhamed et al., 2014). We show that six aquaculture bacterial pathogens (i.e., *Aeromonas hydrophila*, *Vibrio alginolyticus*, *Edwardsiella tarda*, *Streptococcus iniae*, *Vibrio harveyi*, and *Vibrio fluvialis*) in the stationary phase are killed rapidly *in vitro* by gentamicin and neomycin under hypoionic shock conditions. Importantly, we demonstrate the *in vivo* efficacy of the new approach against *A. hydrophila* infections in a zebrafish model.

## Materials and Methods

### Strains, Media, and Reagents

The aquatic bacterial strains used in this study consisted of five Gram-negative bacteria (i.e., *Aeromonas hydrophila*, *Vibrio alginolyticus*, *Edwardsiella tarda*, *Vibrio harveyi*, and *Vibrio fluvialis*) and one Gram-positive strain (i.e., *Streptococcus iniae*). The sources of all aquatic bacterial strains and their relevant characteristics are listed in Supplementary Table S1. Bacteria were cultured in Lysogeny broth (LB) medium at 37°C (*V. alginolyticus*, *E. tarda*, *V. harveyi*, and *V. fluvialis*) or 30°C (*A. hydrophila* and *S. iniae*) in a shaker (220 rpm). The antibiotics used in this study were gentamicin and neomycin. Other chemicals used included carbonyl cyanide *m*-chlorophenyl hydrazone (CCCP) and its analog carbonyl cyanide-trifluoromethoxyphenyl hydrazone (FCCP), as well as eugenol (an anesthetic for zebrafish). The information for the antibiotics and chemicals used in the study is presented in Supplementary Table S2. All chemical reagents were of analytical purity.

### Antibiotic Tolerance Test for Six Aquatic Bacterial Cells in Stationary Phase

In brief, the aquatic bacterial strains from frozen stock listed in Supplementary Table S1 were seeded in LB medium at a ratio of 1:1,000 and cultured at 37°C (*V. alginolyticus*, *E. tarda*, *V. harveyi*, and *V. fluvialis*) or 30°C (*A. hydrophila* and *S. iniae*) in a shaker (220 rpm) for 24 h to prepare stationary-phase cells, as previously described (Yao et al., 2016; Chen et al., 2019). Each antibiotic was added to the cultured cells at varying concentrations (refer to Supplementary Table S2), and the mixture was further agitated for 5 min or 3 h. Hundred microliters of the treated cells was washed twice using phosphate-buffered saline (PBS; 0.27 g/L of KH_2_PO_4_, 1.42 g/L of Na_2_HPO_4_, 8 g/L of NaCl, and 0.2 g/L of KCl, pH = 7.4) with centrifugation (13,000 *g*, 30 s) and spot-plated onto LB agar dishes at a 10-fold serial dilution in PBS. After incubation at 37°C for at least 12 h, the colony-forming units (CFU) on the dishes were counted after taking a picture of each dish.

### Eradication of Aquatic Bacteria by Aminoglycosides Under Hypoionic Shock Conditions

Treatment with aminoglycosides combined with hypoionic shock was performed as previously described (Jiafeng et al., 2015; Chen et al., 2019). In brief, 100 μl of stationary-phase cells was centrifuged (12,000 *g*, 1 min) in an Eppendorf tube, and the medium was completely removed. Cell pellets were re-suspended in ultrapure water (i.e., without the presence of ions; 0.9% NaCl solution was used as the negative control) containing gentamicin or neomycin at the concentrations listed in Supplementary Table S2. Ultrapure water was prepared by Milli-Q^®^ Advantage A10 (Millipore). The cell suspension was kept at room temperature (i.e., 25°C) for 5 min, and the cells were washed twice with PBS before spot-plating on LB agar dishes for cell survival assays as described above. Similarly, the effect of ATP was evaluated by agitating the cell culture in the presence of 20 μM of protonophore CCCP or FCCP for 1 h before the combined treatment.

### Intracellular ATP Level Assay

A luciferase-based kit (BacTiter-Glo^TM^, Promega, G8231) was used to measure ATP levels according to the manufacturer’s instructions. Briefly, stationary-phase cells, with or without 20 μM of protonophore CCCP or FCCP pretreatment for 1 h, were quickly mixed with the working solution at equal volumes and then transferred to a 96-well plate before light recording on a FLUOstar Omega Microplate Reader using a Luminometer.

### Assay for the Uptake of Fluorescent-Labeled Gentamicin

A fluorescent probe (Ex_58__0 *n*__*m*_, Em_600__–__70__0 *n*__*m*_) was attached to gentamicin as we recently reported (Wu et al., 2020). After salts were removed through dialysis, the fluorescent-labeled gentamicin was dissolved in ultrapure water at 100 μg/ml for the treatment of *A. hydrophila* or *V. alginolyticus* cells as described above. The cells were then washed twice with PBS and re-suspended in 500 μl of cell wall-digestion buffer (30 mM of Tris–HCl, pH 8.0, 1 mM of EDTA, 1 mg/ml of lysozyme) for further incubation at room temperature for 5 h. The cells were subjected to three cycles of freezing treatment at −80°C, followed by thermal denaturation at 90°C for 10 min (note: we confirmed that gentamicin had high thermal stability; Chen et al., 2019), and then centrifuged to remove the cell debris and denatured proteins. Afterward, an equal volume of supernatant containing fluorescent-labeled gentamicin was placed in a quartz cuvette, and the fluorescence intensity in the supernatant was determined with a fluorescence spectrometer (Spectrofluorometer FS5, EDINBURGH). In addition, the amount of fluorescent-labeled gentamicin uptaken by CCCP or FCCP-pretreated cells was measured. A standard curve was prepared by directly adding fluorescent-labeled gentamicin at different concentrations (0, 25, 50, and 100 μg/ml) to persister cells suspended in cell wall digestion buffer. All procedures described above were performed under dim lighting to prevent fluorescence quenching.

### Animal Experiments

Zebrafish were purchased from Fuzhou aquarium market and acclimated for 2 weeks before infection. The zebrafish were handled according to the procedures defined by the Animal Ethical and Welfare Committee of Fujian Normal University (approval no. IACUC 20190006, Fuzhou, China). The zebrafish were anesthetized after being immersed in 45 mg/L of eugenol for about 8 min (Sánchez-Vázquez et al., 2011). After anesthesia, the fish were lightly scraped along the lateral surface behind the pectoral fins with a sterile scalpel to remove several scales (Neely et al., 2002; Zhang et al., 2016). When the scraped fish recovered from anesthesia, they were infected by swimming in water containing 5.0 × 10^7^ CFU/ml of *A. hydrophila* at 30°C for 3 h. Zebrafish infected by *A. hydrophila* were rinsed with deionized water for a few seconds and then were randomly divided into three groups (A: mock group without treatment; B: treated with gentamicin-containing ultrapure water; and C: treated with gentamicin-containing 0.9% NaCl solution). The concentration of gentamicin in animal experiments is 25 μg/ml. After treatment for 5 min, three fish from each group were selected randomly, and two-fifths of each fish’s body was cut, weighed, and homogenized in saline at a ratio of 100 mg/ml (w/v). The lysates were spot-plated on LB agar dishes for bacterial survival assays. For quantification, each sample was spot-plated in triplicate. The injury and survival rates of the zebrafish were observed 48 h after antibiotics treatment. All experiments were performed three times. In addition, three fish scraped without infection were homogenized to count the basal number of bacteria in the fish themselves.

## Results

### Five-Minute Hypoionic Shock Treatment Enables Gentamicin and Neomycin to Kill Stationary-Phase Aquaculture Bacteria

**FIGURE 1:**
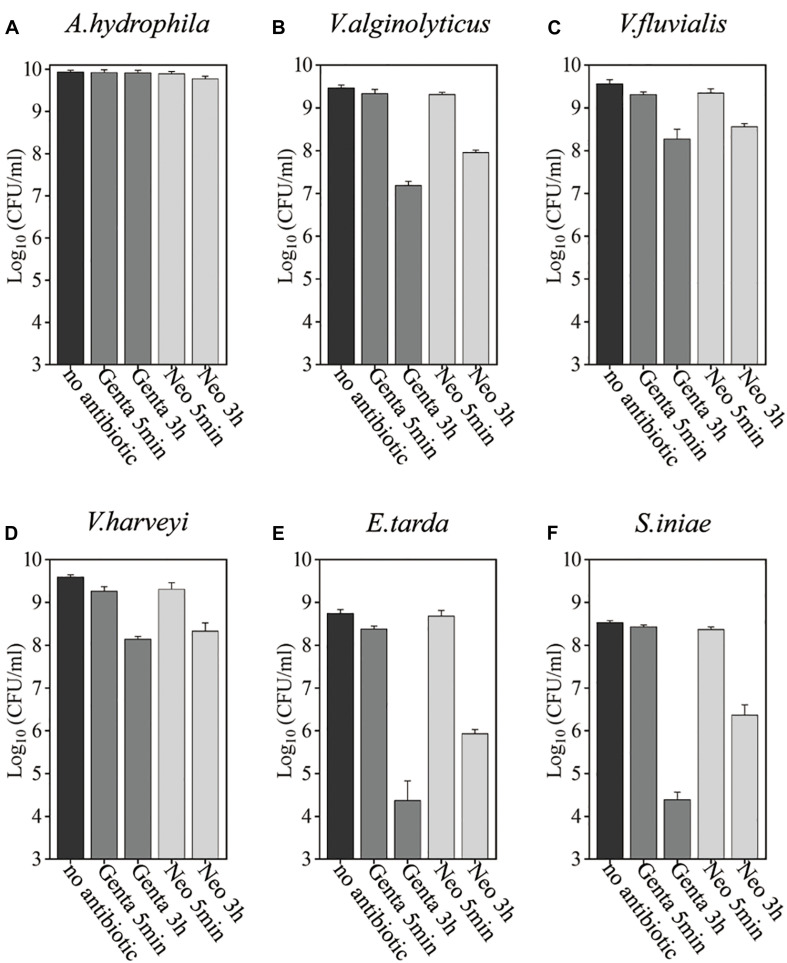
Antibiotic tolerance of six aquaculture bacteria under conventional treatment conditions. **(A–F)** Stationary-phase cells of the indicated bacteria [*Aeromonas hydrophila*, *Vibrio alginolyticus*, *Vibrio fluvialis*, *Vibrio harveyi*, *Edwardsiella tarda*, and *Streptococcus iniae*, **(A–F)**, respectively], which were treated with gentamicin (Genta) or neomycin (Neo) dissolved in 0.9% NaCl solution for 5 min or 3 h and then spot-plated on Lysogeny broth (LB) agar dishes for cell survival assays. The concentrations of antibiotics used were as follows: 25 μg/ml of Genta and 50 μg/ml f Neo for S. iniae; 100 μg/ml f Genta and 200 μg/ml f Neo for the other five bacteria.

Bacteria in the stationary phase are highly tolerant to antibiotics under conventional treatment conditions, presumably due to stress- or starvation-induced growth arrest (Rittershaus et al., 2013; Martins et al., 2018). Here, we examined the antibiotic tolerance of six aquaculture bacteria, specifically *Aeromonas hydrophila*, *Vibrio alginolyticus*, *Vibrio fluvialis*, *Vibrio harveyi*, *Edwardsiella tarda*, and *Streptococcus iniae* (with the concentrations of these cells being 8.6 × 10^9^, 3 × 10^9^, 3.6 × 10^9^, 3.9 × 10^9^, 5.5 × 10^8^, and 3.4 × 10^8^, respectively), by treating the stationary-phase cells with aminoglycoside antibiotics dissolved in 0.9% NaCl solution (Figure 1 and Supplementary Figure S1). In China, gentamicin is widely used, and neomycin is the only aminoglycoside currently certified for aquaculture applications (Liu et al., 2017). Therefore, these two aminoglycosides were evaluated. We found that neither gentamicin nor neomycin could kill the above bacteria after incubation for 5 min when dissolved in 0.9% NaCl solution. When the cells were treated with the antibiotics in 0.9% NaCl solution for 3 h, the bacteria exhibited different degrees of antibiotic susceptibility: 3.4 and 31.3% of *A. hydrophila* were killed by gentamicin and neomycin, respectively; *V. alginolyticus*, *V. fluvialis*, and *V. harveyi* were eliminated by 1∼2 orders of magnitude; *E. tarda* and *S. iniae* were killed by gentamycin by about 4 orders of magnitude.

**FIGURE 2:**
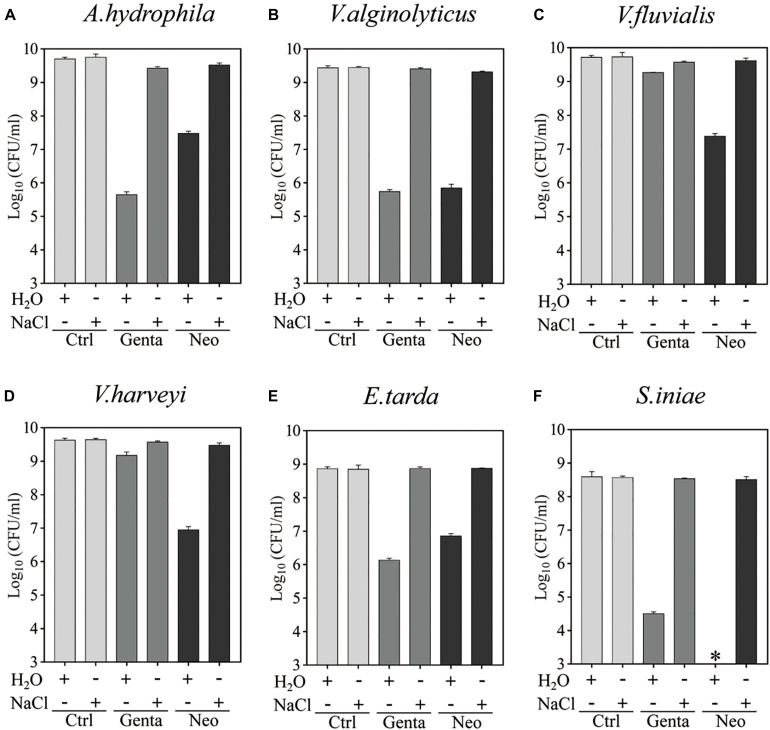
Hypoionic shock-induced potentiation of gentamicin and neomycin effects against the six aquaculture bacteria. **(A–F)** Survival of stationary-phase cells of *Aeromonas hydrophila* **(A)** *Vibrio alginolyticus* **(B)**, *Vibrio fluvialis* **(C)**, *Vibrio harveyi* **(D)**, *Edwardsiella tarda* **(E)**, and *Streptococcus iniae* **(F)** following 5 min treatment with Genta or Neo dissolved in ultrapure water or 0.9% NaCl. The concentrations of antibiotics used were as follows: 25 μg/ml of Genta and 50 μg/ml of Neo for S. iniae; 100 μg/ml of Genta and 200 μg/ml of Neo for the other five bacteria. ^∗^in Panel F indicates that No CFU detected during cell survival assay by 100000-fold dilution.

Bacterial cells in the stationary phase were next treated with gentamicin- or neomycin-containing ultrapure water for 5 min for comparison with the antibiotic treatment in 0.9% NaCl solution (Figure 2 and Supplementary Figure S2). Substantial amounts of all aquaculture bacteria were killed by neomycin-containing ultrapure water. Similarly, *S. iniae* colonies were undetectable on LB agar dishes after 5 min treatment with neomycin-containing ultrapure water (as indicated by the asterisks in Figure 2E), indicating a reduction in the surviving cells by more than 6 orders or magnitude. Notably, *A. hydrophila*, *V. alginolyticus*, *E. tarda*, and *S. iniae* were killed by gentamicin-containing ultrapure water by 3∼4 orders of magnitude, but only a very limited potentiation effect was observed on gentamicin with *V. fluvialis* and *V. harveyi* (Supplementary Figure S2B). In contrast, neither gentamicin nor neomycin had a killing effect when dissolved in the NaCl solution. These results indicate that hypoionic shock treatment enables aminoglycoside antibiotics to rapidly reduce stationary-phase aquaculture bacteria.

### Hypoionic Shock-Induced Gentamicin Potentiation Against Aquaculture Bacteria Is Partially Dependent on ATP

The tolerance of bacteria to antibiotics is closely related to intracellular ATP levels (Meylan et al., 2017; Shan et al., 2017; Pu et al., 2019). We next sought to determine whether hypoionic shock-induced aminoglycoside potentiation against aquaculture bacteria is affected by the ATP level in the bacterial cells. CCCP and FCCP are uncouplers of the proton motive force (PMF) that drives ATP synthesis and thus are able to reduce intracellular ATP levels (Kinoshita et al., 1984; Tapia et al., 2006). To this end, we treated aquaculture bacteria (*A. hydrophila*, *V. alginolyticus*, *E. tarda*, and *S. iniae*) with CCCP and FCCP for 1 h and then subjected them to gentamicin treatment under hypoionic shock conditions (note: *V. fluvialis* and *V. harveyi* were not analyzed here due to a very limited potentiation effect of hypoionic shock on gentamicin).

**FIGURE 3:**
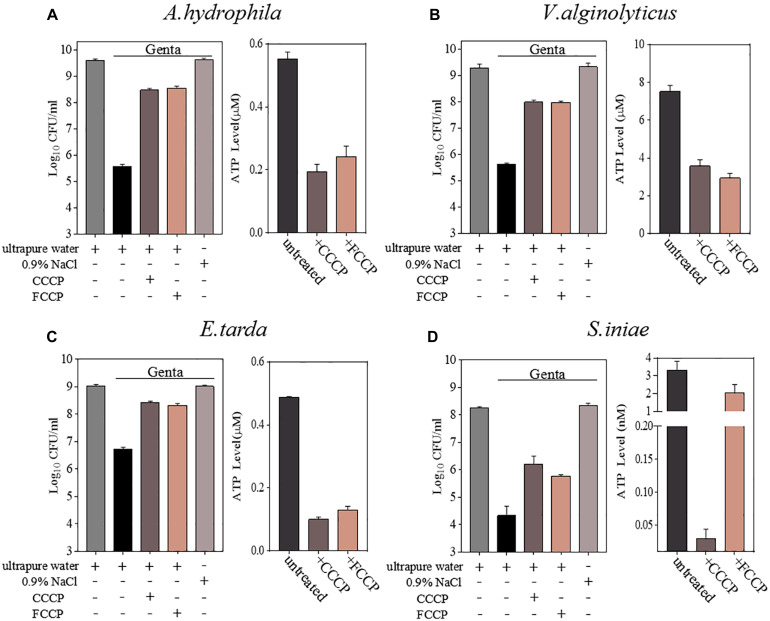
Hypoionic shock-induced potentiation of gentamicin effects against aquatic pathogenic bacteria is partially dependent on ATP. **(A–D)** Left parts in each panel: survival of stationary-phase cells of *Aeromonas hydrophila*, *Vibrio alginolyticus*, *Edwardsiella tarda*, and *Streptococcus iniae*. Cells were pretreated with CCCP or FCCP for 1 h and then subjected to 5-min treatment with gentamicin dissolved in ultrapure water. Right parts in each panel: ATP levels in CCCP- or FCCP-pretreated cells.

Cell survival assays revealed that CCCP, as well as its functional analog FCCP, efficiently suppressed the hypoionic shock-induced gentamicin potentiation that kills stationary-phase aquaculture bacteria in 5 min (Figure 3 and Supplementary Figure S3). An intracellular ATP assay also confirmed that pretreatment with CCCP or FCCP significantly reduced the intracellular ATP levels in all of the bacteria evaluated (Figure 3). These results suggest that hypoionic shock-induced potentiation of gentamicin’s effect against aquaculture bacteria is, at least, partially dependent on intracellular ATP.

### Hypoionic Shock Enhances the Bacterial Uptake of Gentamicin

**FIGURE 4:**
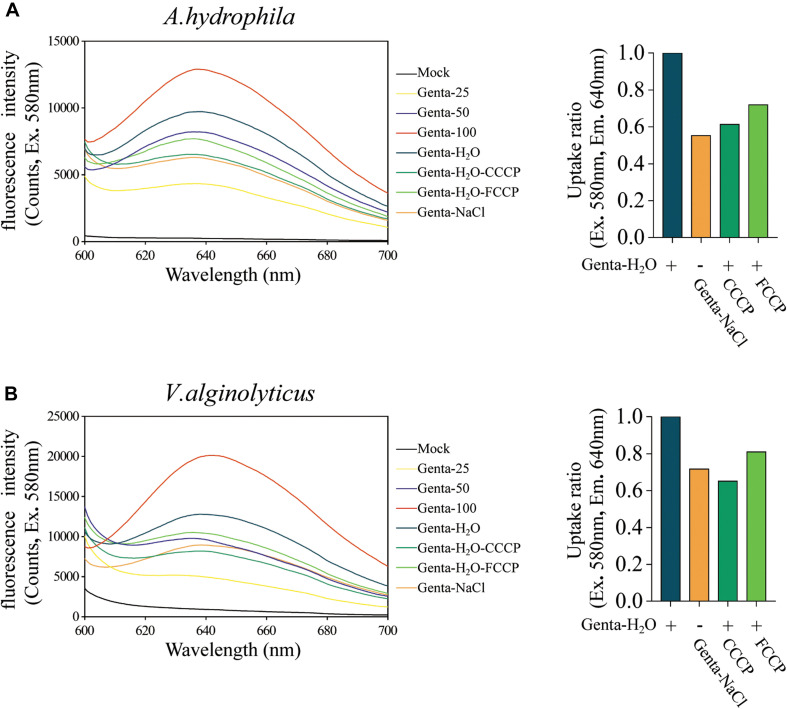
Hypoionic shock enhances the bacterial uptake of fluorescent-labeled gentamicin. **(A,B)** Left parts: the fluorescence spectra for fluorescent gentamicin taken up by *Aeromonas hydrophila* **(A)** or *Vibrio alginolyticus* **(B)** were monitored with a spectrofluorometer. Mock, bacteria lysate without antibiotic treatment. Genta 25–100, standard concentration fluorescence labeled antibiotic (25, 50, and 100 μg/ml) mixed with bacteria lysate. In the Genta-H_2_O (Genta dissolved in ultrapure water), Genta-NaCl (Genta dissolved in saline), Genta-H_2_O-CCCP (CCCP-pretreated and Genta dissolved in ultrapure water), and Genta-H_2_O-FCCP (FCCP-pretreated and Genta dissolved in ultrapure water) groups, the fluorescent gentamicin taken up by bacterial cells was monitored with a spectrofluorometer. **(A,B)** Right parts: ratios for fluorescent gentamicin uptake in different experimental groups compared with those in the hypoionic shock-treated group (Genta-H_2_O).

Aminoglycoside antibiotics such as gentamicin and tobramycin must traverse the bacterial cytoplasmic membrane prior to initiating their lethal effects, and the uptake of aminoglycosides is facilitated by the PMF or ATP (Taber et al., 1987; Fraimow et al., 1991; Allison et al., 2011). Therefore, we further explored whether hypoionic shock treatment affects the bacterial uptake of gentamicin antibiotics. For this purpose, fluorescent gentamicin was synthesized by conjugating coumarin–hemicyanine scaffolds to gentamicin (Wu et al., 2020). The conjugation did not affect the bactericidal efficacy of gentamicin (Supplementary Figure S4A). The fluorescent gentamicin taken up by *A. hydrophila* or *V. alginolyticus* was extracted through cell wall digestion coupled with cycled freezing/thawing and thermal denaturation and then subjected to a fluorescence assay. Fluorescent gentamicin at standard concentrations was directly incubated with bacterial lysates before a fluorescence assay. The maximal fluorescent intensity of fluorescent gentamicin was approximately 640 nm (Figure 4A).

A regression analysis (Supplementary Figure S4B) based on the standards (Figure 4A) showed that the concentration of gentamicin extracted from *A. hydrophila* upon hypoionic shock was approximately 69.58 μg/ml, while that extracted from the cells treated with 0.9% NaCl solution was only 37.96 μg/ml. Notably, we found that the amount of gentamicin taken up by the cells pretreated with CCCP or FCCP was reduced to 60–70%. Similarly, the concentration of gentamicin extracted from *V. alginolyticus* upon hypoionic shock was about 64.45 μg/ml, which was significantly reduced in NaCl solution or upon pretreatment with CCCP or FCCP (Figure 4B).

### Gentamicin Under Hypoionic Shock Conditions Significantly Improved the Survival of Zebrafish Infected by *Aeromonas hydrophila*

In recent years, zebrafish (*Danio rerio*) has been used as an important alternative to mammalian models in the study of human infectious disease (Allen and Neely, 2010; Meijer and Spaink, 2011; Sullivan et al., 2017). In order to explore the *in vivo* efficacy of combined treatment with hypoionic shock and also probe the potential application of the combined treatment against aquaculture bacterial pathogens, a zebrafish infection model was used in this study. After infection by *A. hydrophila* (for details, refer to the “Materials and Methods” section), the zebrafish were rinsed with deionized water for a few seconds and randomly divided into three groups before being subjected to different treatments for 5 min: mock treatment, gentamicin dissolved in ultrapure water (Genta + H_2_O), and gentamicin dissolved in NaCl solution (Genta + NaCl).

**FIGURE 5:**
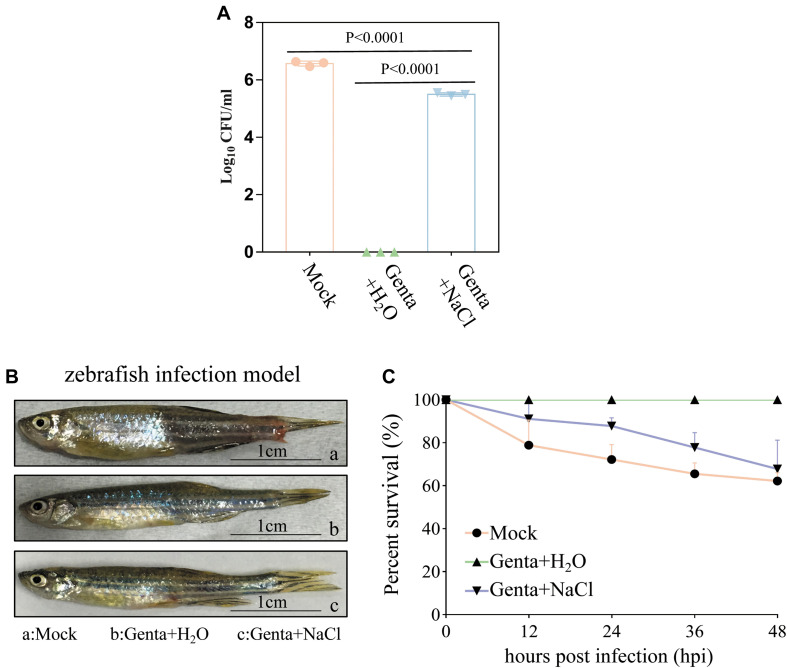
Hypoionic shock facilitates gentamicin killing of *Aeromonas hydrophila* in a zebrafish model. **(A)** The survival percentage of the pathogenic bacteria on/in the zebrafish treated with gentamicin dissolved in ultrapure water (Genta + H_2_O) or in 0.9% NaCl solution (Genta + NaCl) for only 5 min. The mock groups were not treated with antibiotics. The number of bacteria on/in the zebrafish before infection was subtracted (Supplementary Figure S5C) from the results for each group. **(B)** The gross pathology of zebrafish infected by *A. hydrophila* at 12 h. The infection site of some fish in the mock groups and salt treatment groups became red and swollen. **(C)** The survival percentage of zebrafish infected by *A. hydrophila* after 48 h.

Bacterial cell survival assays revealed that 3.75 × 10^6^ CFU/ml of *A. hydrophila* cells from the homogenate of wound tissues from zebrafish were viable without treatment (mock). Treatment with Genta + NaCl reduced the number of viable cells to 3.3 × 10^5^ CFU/ml. Strikingly, viable *A. hydrophila* cells were almost undetectable when the zebrafish were treated with Genta + H_2_O (Figure 5A). These results indicated that short-term exposure to gentamicin-containing ultrapure water is more effective at killing bacteria in infected fish than exposure to gentamicin-containing NaCl solution.

Consistently, we observed that the infection sites in some fish in the mock group and Genta + NaCl group became red and swollen 12 h after infection (Figure 5B). Animal survival assays revealed that approximately 20% of zebrafish in the control (mock) group began to die 12 h post-infection, and only about 60% of this group survived 48 h post-infection. In the Genta + NaCl group, 70% of zebrafish survived 48 h post-infection. Surprisingly, none of the zebrafish in the Genta + H_2_O group died or exhibited swelling (Figure 5C).

## Discussion

Outbreaks of infectious disease are considered a significant constraint in the aquaculture industry, causing more than 10 billion USD worth of losses annually on a global scale (Evensen, 2016). The currently available commercial vaccines are aimed at specific animals, for example, some fish and lobsters (Assefa and Abunna, 2018; Adams, 2019), and cannot be widely administered to other aquatic animals, especially invertebrates lacking acquired immunity. Most of the novel alternative biocontrol strategies for fish bacterial diseases, such as probiotics, bio-encapsulated vaccines, and phage therapy, are still in the research phase (Pérez-Sánchez et al., 2018; Soliman et al., 2019). On the contrary, the activity spectrum, mode of action, resistance mechanisms, and current applications of most important antibiotic classes have been well investigated (Mohr, 2016). It is highly desirable to develop more effective and safer approaches for current antibiotics. For example, Peng et al. (2015) found that exogenous glucose or alanine plus kanamycin can kill multidrug-resistant *Edwardsiella tarda* both *in vitro* and in a mouse model for urinary tract infection. Glycerol monolaurate, lauric acid, 5-methylindole, and even the commonly used diabetic drug metformin were found to act synergistically with aminoglycoside to eliminate *Staphylococcus aureus* persisters (Hess et al., 2014; Liu et al., 2020; Sun et al., 2020). Various adjuvants, including metabolites (Allison et al., 2011; Peng et al., 2015), for antibiotic potentiation have been well documented (Liu et al., 2019).

Here, we show that hypoionic shock enables gentamicin and/or neomycin antibiotics to reduce six stationary-phase aquaculture pathogenic bacteria, consistent with our previous studies (Jiafeng et al., 2015; Chen et al., 2019). Furthermore, this hypoionic shock-induced potentiation of gentamicin was also observed in zebrafish infected with *Aeromonas hydrophila*. Mechanistically, the potentiation seems to be achieved by enhancing the bacterial uptake of gentamicin under hypoionic shock conditions. Given pathogenic bacteria generally infect fish on the surface of the skin (Benhamed et al., 2014), our approach may represent a promising strategy for bacterial infection control in aquaculture. This approach is eco-friendly, non-toxic, and non-immunogenic, with the exception of the required operation to transfer the fish into aminoglycoside-containing ultrapure water. Currently, a small-scale application of this approach is being performed in our laboratory.

Our approach is also advantageous with respect to food safety and environmental health, given that it takes only a few minutes to complete the treatment; therefore, a smaller amount of antibiotics and less time for antibiotic exposure are required. As such, contamination of the environment by residual antibiotics and/or retention of the antibiotics within animal bodies can be minimized. Currently, the use of antibiotics in aquaculture leads to the accumulation of residual antibiotics in sea and freshwater foods (Song et al., 2017; Chen et al., 2018), which may have adverse effects on humans (Cabello, 2006). Similarly, residual antibiotics in water or aquatic sediments may facilitate the spread of antibiotic resistance in environmental bacteria (Gullberg et al., 2011; Cabello et al., 2016). In addition, compared with the stimulation of aminoglycoside potentiation by metabolites, which takes a few hours to manifest (Allison et al., 2011; Peng et al., 2015), our approach, which does not consume metabolites and requires only a few minutes, may have certain advantages.

## Data Availability Statement

The original contributions presented in the study are included in the article/Supplementary Material, further inquiries can be directed to the corresponding author/s.

## Ethics Statement

The animal study was reviewed and approved by the Animal Ethical and Welfare Committee of Fujian Normal University (approval no. IACUC 20190006, Fuzhou, China).

## Author Contributions

XF and YG designed the study. ZC and WY performed the experiments. DL synthesized the fluorescent gentamicin. YG and XF analyzed the data and wrote the manuscript. All authors contributed to the article and approved the submitted version.

## Conflict of Interest

The authors declare that the research was conducted in the absence of any commercial or financial relationships that could be construed as a potential conflict of interest.
