# Supplementary material for: Gentamicin Combined With Hypoionic Shock Rapidly Eradicates Aquaculture Bacteria in vitro and in vivo
Source: Front Microbiol. 2021 Apr 6;12:641846. doi: 10.3389/fmicb.2021.641846 (PMC8055967; doi:10.3389/fmicb.2021.641846)
Supplement: Supplementary file 1 [file Table_1.DOCX]

### Supporting information file

**Gentamicin combined with hypoionic shock rapidly reduces aquaculture bacteria *in vitro* and *in vivo***

Yuanyuan Gao, Zhongyu Chen, Wei Yao, Daliang Li and Xinmiao Fu

### Table S1 Bacterial strains used in this study

| **No** | **Bacterial strains** | **Origins** | **Characteristics** |
| --- | --- | --- | --- |
| 1 | *Aeromonas hydrophila*  ATCC7966 | Our laboratory. | G^-^ |
| 2 | *Edwardsiella tarda*  ATCC1594 | A gift from Dr. Chao Wang at Shandong Freshwater Fisheries Research Institute | G^-^ |
| 3 | *Vibrio harveyi* | A gift from Prof. Yuelin Zhang at Shantou University | G^-^ |
| 4 | *Vibrio fluvialis* |  | G^-^ |
| 5 | *Vibrio alginolyticus* |  | G^-^ |
| 6 | *Streptococcus iniae* |  | G^+^ |

### Table S2 Antibiotics and reagents used in this study

| **No** | **Antibiotics** | **Suppliers** | **concentration** |
| --- | --- | --- | --- |
| 1 | Gentamicin (Genta) | Sangon Biotech (Shanghai) Co., Ltd. | 25 μg/mL for *S. iniae*; 100 μg/mL for other five bacteria |
| 2 | Neomycin (Neo) | Sangon Biotech (Shanghai) Co., Ltd. | 50 μg/mL for *S. iniae*; 200 μg/mL for other five bacteria |
| 3 | Fluorescence-labeled Genta | Made in our laboratory [^1^](#_ENREF_1) | 25, 50, 100 μg/mL for uptake assay |
| 4 | NaCl | Transgen Biotech Co., Ltd. | 0.9% (w/v) |
| 5 | Carbonyl cyanide-  chlorophenyl hydrazone (CCCP) | Sigma-Aldrich Co., Ltd. | 20 μM |
| 6 | Carbonyl cyanide-p-  trifluoromethoxyphenyl hydrazone (FCCP) | Sigma-Aldrich Co., Ltd. | 20 μM |
| 7 | Eugenol | Sigma-Aldrich Co., Ltd. | 45 mg/L |
| 8 | Lysozyme | Beijing Solarbio Science & Technology Co., Ltd | 1 mg/mL |

**Figure S1**

### Fig. S1 Antibiotic tolerance test of six aquaculture bacteria under conventional treatment conditions

(**A-F**) Survival of stationary-phase cells of *A. hydrophila*, *V. alginolyticus*, *V. fluvialis*, *V. harveyi*, *E. tarda* and *S. iniae* were treated with gentamicin (Genta) or neomycin (Neo ) in 0.9% NaCl solution for 5 min or 3 h. The antibiotics concentrations used: 25 μg/mL Genta and 50 μg/mL Neo for *S. iniae*; 100 μg/mL Genta and 200 μg/mL Neo for the other bacteria.

**Figure S2**

### Fig. S2 Hypoionic shock promotes aminoglycosides to kill several aquatic bacterial persister cells.

**(A)** Survival of stationery-phase cells following 5-min treatment with Genta or Neo in ultrapure water or NaCl solution. The concentrations of antibiotics used are as follows: *S. iniae* (Genta: 25 μg/mL, Neo: 50 μg/mL); the other five bacteria (Genta: 100 μg/mL, Neo: 200 μg/mL). (**B**) Survival of stationery-phase cells of *V. fluvialis* and *V. harveyi* after the following treatments: G-200: 200 μg/mL gentamicin in ultrapure water or NaCl solution, 5 min; G-100: 100 μg/mL gentamicin in ultrapure water or NaCl solution, 10 min.

**Figure S3**

### Fig. S3 CCCP or FCCP pretreatment suppressed hypoionic shock-induced gentamicin potentiation

(**A-D**) Survival of stationary-phase cells of *A. hydrophila* (panel A), *V. alginolyticus* (panel B), *E. tarda* (panel C) and *S. iniae* (panel D) following a 5-min treatment with Genta dissolved in ultrapure water or in saline solution. Cells were pretreated with CCCP or FCCP for 1 h (refer to **Fig. 3**)

**Figure S4**

### Fig. S4 Bacterial uptake of fluorescent labeled gentamicin

(**A**) Survival of stationary-phase cells of the indicated bacteria following 5-min treatment with fluorescent labeled gentamicin dissolved in ultrapure water or NaCl solution. The addition of fluorescent probe does not affect the germicidal efficacy of gentamicin. Fluorescent antibiotics were synthesized by conjugating coumarin hemicyanine scaffolds to gentamicin. (**B**) Regression analysis based on fluorescent intensity of standard concentration fluorescent gentamicin at 640 nm (refer to **Fig. 4**). Fluorescent gentamicin at standard concentrations was directly added into the lysozyme-containing, cell wall-digestion buffer at indicated concentration.

**Figure S5**

### Fig. S5 Survival of *A. hydrophila* in infected zebrafish

(**A**) Survival of *A. hydrophila* treated with gentamicin dissolved in ultrapure water or in saline solution for 5 min *in vitro*. (**B, C**) Survival of *A. hydrophila* in the infected zebrafish after the combined treatment. The experimental groups were treated with gentamicin dissolved in ultrapure water (Genta+H_2_O) or in saline solution (Genta+NaCl) for only 5 min. The zebrafish of mock groups were not treated with antibiotics. The zebrafish of “self” groups were not infected intentionally with any bacteria.

1. Wu L., Chen L., Kou M., Dong Y., Deng W., Ge L., Bao H., Chen Q., and Li D. ( 2020). The ratiometric fluorescent probes for monitoring the reactive inorganic sulfur species (RISS) signal in the living cell. *Spectrochim Acta A Mol Biomol Spectrosc.* 15;231:118141. doi: 10.1016/j.saa.2020.118141.
